# Transcriptomic Analysis Links Eosinophilic Esophagitis and Atopic Dermatitis

**DOI:** 10.3389/fped.2019.00467

**Published:** 2019-11-20

**Authors:** Rémi Doucet-Ladevèze, Sébastien Holvoet, Frédéric Raymond, Francis Foata, Gurjit K. Khurana Hershey, Joseph D. Sherrill, Marc E. Rothenberg, Carine Blanchard

**Affiliations:** ^1^Nestlé Institute of Health Sciences, Nestlé Research, Lausanne, Switzerland; ^2^Division of Allergy and Immunology, Department of Pediatrics, Cincinnati Children's Hospital Medical Center, University of Cincinnati College of Medicine, Cincinnati, OH, United States; ^3^Division of Asthma Research, Department of Pediatrics, Cincinnati Children's Hospital Medical Center, University of Cincinnati College of Medicine, Cincinnati, OH, United States

**Keywords:** asthma, atopic dermatitis, eosinophilic esophagitis, epithelial cells, interleukin 13

## Abstract

**Background:** Eosinophilic esophagitis (EoE) is commonly associated with concomitant atopic diseases including atopic dermatitis (AD) and allergic airway (AA) diseases including asthma. Despite this link and the shared pathologic features across these three disorders, detailed analyses of the unifying molecular pathways are lacking.

**Objectives:** We sought to investigate the mRNA expression profile overlap between EoE, AA, and AD and to identify the involvement of interleukin 13 (IL-13) in modulating gene expression.

**Methods:** Whole-genome mRNA expression analyses were performed on tissue specimens (biopsies or nasal brushes) from patients with EoE, AD, and AA, and IL-13-stimulated primary human epithelial cells from the esophagus, the skin, and the airways.

**Results:** By human disease expression profiles, EoE evidenced a significantly higher overlap (*p* = 0.0006) with AD (181 transcripts; 10%) than with AA (124 transcripts, 7%). Only 18 genes were found to be commonly dysregulated among the three diseases; these included filaggrin, histamine receptor H1, claudin 1, cathepsin C, plasminogen activator urokinase receptor, and suppressor of cytokine signaling 3. Ontogenetic analysis revealed a common immune/inflammatory response among the three diseases and a different epithelial response (epidermal cell differentiation) between EoE and AA. The overlap between the IL-13–stimulated epithelial cell transcriptome and the respective disease transcriptome was 22, 9, and 5% in EoE, AD, and AA, respectively, indicating a greater involvement of the IL-13 pathway in EoE than AA (*p* = 0.0007) or AD (*p* = 0.02).

**Conclusion:** EoE, AD, and AA share a common set of disease-specific transcripts, highlighting common molecular etiology. Their comparative analysis indicates relatively closer relationships between EoE and AD, particularly centered around IL-13–driven pathways. Therefore, these findings provide an increased rationale for shared therapeutic strategies.

## Introduction

Over the past several decades, the occurrence of allergic diseases has been increasing worldwide. Allergic diseases are chronic and debilitating disorders that may progress into life-threatening conditions (1). The European Association of Allergy and Clinical Immunology recently estimated that one in four school-age children suffer from allergy (2, 3). It is estimated that 150 million individuals in Europe alone suffer from a form of allergy. Food allergy affects up to 8% of children, whereas allergic airway disease (AA), such as asthma, affects up to 20% of children (1–3). The prevalence of atopic dermatitis (AD) varies considerably geographically but can affect up to 20% of children in some countries (1–3). Eosinophilic esophagitis (EoE) is an emerging, antigen-driven esophageal inflammatory disease characterized by eosinophilic infiltration into the esophageal mucosa. Still a rare disease, EoE may be underdiagnosed, and its prevalence is estimated at 0.5–1 in 2000 (4).

Previous reports have highlighted the association of EoE with a concurrent or historic diagnosis of AD or AA (5–8). Additionally, preclinical studies have also shown that epicutaneous sensitization can prime the esophagus to recruit eosinophils in the lungs and in the esophagus after an intranasal challenge with the allergen (9), linking the three diseased organs: the skin, the esophagus, and the lungs (9, 10). Of note, the epithelial architecture of the esophagus and the skin, though originating from different germ layers, have both converged to a stratified, squamous morphology (although human esophageal epithelium lacks a cornified layer). Arora and Yamazaki suggested that EoE might be “the asthma of the esophagus” (11), and several *in vivo* models have highlighted the remarkable link between allergic airway inflammation and the presence of eosinophils in the esophagus (9, 12, 13), suggesting a common etiology and possibly common predisposing factors. Genetic analyses have identified disease-specific and common genetic predisposing factors to EoE, AD, and AA. Polymorphisms in *TSLP* (thymic stromal lymphopoietin) and loss-of-function mutations in the epithelial differentiation gene *FLG* (filaggrin) have been shown to contribute to the occurrence of AD and EoE, whereas a *CCL26* (eotaxin-3) polymorphism is associated with EoE and AA (14–21). The risk locus 11q13, encoding the genes *EMSY* and *LRRC32*, has been associated with EoE (22), AD (23), and AA (24); other variants, such as in *CAPN14*, are more specific for EoE, as *CAPN14* is specifically expressed in esophageal epithelial cells (14, 25). The age of onset of EoE, AD (26), and AA (27) may also be associated with genetic variants; however, as this knowledge is preliminary in AA and AD and absent in EoE, commonalities across these diseases are not known. Another key overlapping feature between the three Th2 diseases is that EoE, AD, and AA are all treated with topical corticosteroids, though delivery is swallowed (topical), epicutaneous, and nasal/inhaled for EoE (4), AD (28), and AA (29), respectively.

EoE, AD, and AA are characterized by Th2-associated inflammation marked by the production of Th2 cytokines, such as IL-4/IL-13, and the recruitment of Th2 cells into the inflamed tissues. IL-13 has been largely shown to be involved in EoE (13), AD (9, 10, 30), and AA (31) pathogenesis in *in vivo* models and genetically modified animals. IL-13 is responsible for the transcription of numerous genes in Th2 inflamed tissues. Moreover, we have previously demonstrated that IL-13 was involved in the gene expression profile observed in esophageal biopsies derived from patients with EoE (32). IL-13 has been shown to induce the expression of numerous genes, such as the eosinophil chemoattractants, the eotaxins, by epithelial cells of the lung, esophagus, and skin. However, the subtype induction of eotaxin-1, 2, or 3 is tissue-specific (20, 33) in these diseases. Numerous other inflammatory mediators induced by IL-13 may also be tissue, cell, or species-specific or observed only *in vitro*. The direct comparison of the expression profiles of Th2 diseases with their respective IL-13–stimulated epithelial cell and murine model expression profiles may highlight the tissue and species specificities of these inductions. Human clinical trials testing the efficacy of blocking the IL-4/IL-13 pathway in the three diseases have provided positive results, and anti–IL-4α, which blocks IL-4 and IL-13, is already approved for AD and AA (34–36). Understanding the involvement of IL-13–induced expression changes in these diseases is thus critical to better understand the trial outcomes.

Transcriptomic analyses have been conducted on inflamed tissue specimens derived from patients with EoE, AD, and acute asthma, providing molecular insights in disease pathogenesis (37–43). The AA transcriptome was described in several human and murine studies and highlighted a large magnitude of genes involved in AA (37–40). The EoE transcriptome analyses have identified the dysregulation of more than 500 genes in esophageal biopsies and hundreds in murine models (41, 42). Finally, numerous studies have analyzed the gene expression profile of non-affected and affected AD skin in humans (44) and mice (43), but few have compared the disease to the respective *in vivo* models (43). These studies are usually followed by candidate-gene approaches in efforts to assign functional importance with respect to disease pathogenesis *in vivo* in mice. Similarly, microarray analysis of atopic skin has led to the identification of *CXCR3, FLG*, and *TARC* in AD and *IL13*, eotaxins, periostin (*POSTN*), and several other genes and cytokines in EoE (20, 45, 46). It is interesting to note that eotaxin-3, though being a top gene upregulated in EoE and being highly induced by IL-13 *in vitro*, seems to be a pseudogene in mice; eotaxin-1 and eotaxin-2 are more paramount in mice (42). Overall, the candidate-gene analyses of these Th2 diseases have highlighted both similarities and some discrepancies among the molecular pathogeneses of EoE, AD, and acute asthma.

Therefore, our overall aim was to develop a comprehensive transcriptomic understanding of three Th2 diseases (EoE, AD, and AA) across three different model systems: inflamed tissues from patients with EoE, AD, or AA; primary epithelial cells stimulated with IL-13 *in vitro*; and tissues from murine models of each disease. We identified both known and novel Th2-induced molecules in the epithelial cells from three different compartments (esophagus, skin, airways) and examined the expression level of the relevant genes in human diseased tissues. IL-13–regulated epithelial cell genes appeared to be represented in all three diseases but significantly differed between the diseases or models. We also identified genes that were dysregulated in the three diseases but were not necessarily dysregulated similarly. Finally, we uncovered a significantly increased overlap of EoE and AD (vs. EoE-AA overlap), suggesting that EoE shares more similarity to AD than AA, specifically in regard to the epithelial gene response.

## Methods

### *In vitro* Models

A pool of primary human epidermal cells derived from the skin (HPEKp, CELLnTEC or C-001-05C, Gibco) were cultivated *in vitro* in 12-well plates in CnT57 medium (CELLnTEC). Once confluent, 10% fetal calf serum was added to the medium for 24 h; cells were stimulated for 48 h with 0 or 100 ng/mL IL-13 (#200-13, Peprotech). The supernatant was removed, and adherent cells were washed with a saline buffer and then frozen at −80°C until RNA extraction. For the transcriptome of IL-13–stimulated airway epithelial cells, expression data from human nasal epithelial cells was used (GSE19182). The human esophageal primary keratinocyte transcriptomes were also derived from human primary esophageal epithelial cells stimulated 48 h with IL-13 as previously published (GSE8853).

### Human Diseases

Data sets were previously published (32) or obtained from public databases (www.ncbi.nlm.nih.gov/pubmed) GEO repository [GSE32924 (47) and GSE19187]. For human AA, the transcriptome from nasal brushes from patients with acute asthma was used and compared to patients without asthma. For EoE disease, esophageal biopsies from patients with EoE and without EoE [normal (NL)] were used (32). For AD, data were generated from punch biopsies in patients with and without AD (47). To ensure comparability, the microarray platform used and the age of the data set were both selected to be similar.

### Murine Models

For the AD model, 5–7–weeks-old female BALB/c J mice (Charles River, L'Arbresle, France) were sensitized by two, successive epicutaneous applications with 200 μg of *Aspergillus fumigatus* (Greer). A small part of the back of the mouse was shaved while it was under isoflurane anesthesia. A sterile gauze (1 × 1 cm) soaked with the allergen was secured to the skin with a bio-occlusive transparent dressing (#2461, Johnson and Johnson) and a band-aid. The patch remained on the skin for a sensitization period of 7 consecutive days. Fourteen days later, a second epicutaneous sensitization was performed. Mice were scored on the basis of skin symptoms. Transepidermal water loss (TEWL) was measured on the sensitization site using a Tewameter® instrument TM300 from Courage + Khazaka Electronic, Köln. Mice were euthanatized 72 h after the second patch removal. Pieces of skin were kept at 4°C in RNALater (Qiagen) until RNA extraction. This model was reviewed and authorized by the “Office Véterinaire du Canton de Vaud.” For the AA inflammation model, the lung transcriptome from mice subjected to repeated intranasal challenges with *Aspergillus fumigatus* extract was used as previously described (48). For the EoE model, the esophageal transcriptomes from the double transgenic mice CC10-rtta /Tet-on-*Il13* mice treated with or without doxycycline were used as previously described (42).

### RNA Extraction

Total RNA was extracted from the skin with RNeasy mini kit (Qiagen) or from the primary cell culture RNAdvance Tissue kit (Agencourt), following the manufacturer's protocol. The extraction quality of RNA was assessed (RNA 6000 Nano kit, Agilent), and a minimum RNA integrity number (RIN) score of seven was the threshold for performing microarray analysis.

### Microarray

Samples were prepared for the microarray per the supplier's protocol (3′ IVT express kit, Affymetrix). Hybridization to the chip (Affymetrix Gene Chip Mouse genome 430 2.0 or Affymetrix Gene Chip Human Genome U133 plus 2.0) was performed following the supplier's information. All data were analyzed with the Genespring GX software (Agilent Technologies). Each data set was normalized using the Robust Multi-array Average (RMA) method (49). Data in the graphs are presented as fold change normalized to the median of the control samples. Ontology analysis was performed using “The Database for Annotation, Visualization and Integrated Discovery” (DAVID) (50).

### Statistics

Differentially expressed genes were identified on the basis of fold change and Welch's *t*-test comparison between the samples and their related negative control. False discovery rate (FDR) correction with corrected *p* ≤ 0.05 was always employed in human disease analyses because the sample size was sufficient. Genes identified by different algorithms are shown in Supplemental Data File 1. The three Th2 disease transcriptomes were compared to the respective IL-13–stimulated primary cells and *in vivo* model. The detection of human and mouse homologs (similarity attributable to descent from a common ancestor) among the annotated genes of several completely sequenced eukaryotic genomes was performed using a HomoloGene table ftp://ftp.ncbi.nih.gov/pub/HomoloGene in the Genespring GX software. Each dataset was first analyzed separately. In order to overcome the bias linked with (1) the different level of dysregulated genes in the different models (Supplementary Figure 1), and (2) the difference of power of the statistical tests due to the difference in the number of samples between diseases, we generated, and compared gene lists of similar sizes containing only significantly dysregulated genes (*p* < 0.05). Enrichment for overlapping genes between different transcriptome comparisons was analyzed using a two-sided Chi-square test or Fisher exact test when the number of events was below 10.

## Results

### Greater Overlap of EoE and AD Transcriptomes Than EoE and AA Transcriptomes

**Figure 1:**
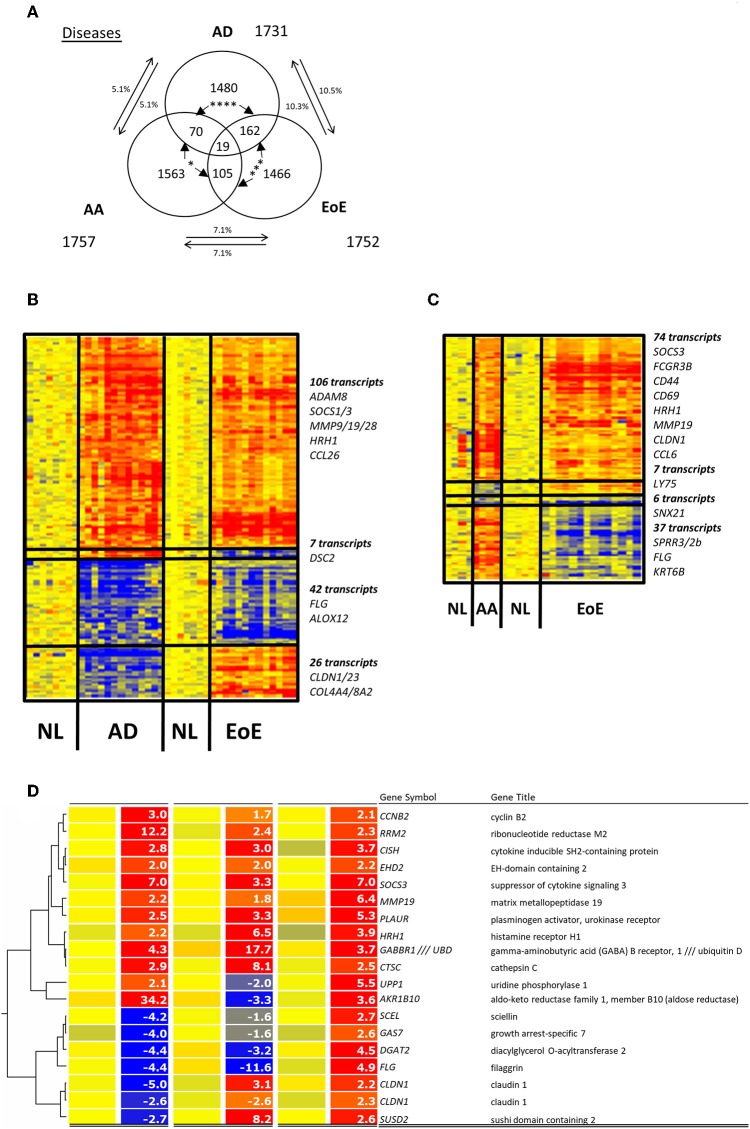
Comparison of EoE, AD, and AA diseases. **(A)** Venn diagram of dysregulated transcripts in the three diseases is shown. The three amounts of dysregulated genes in EoE, AD, and AA diseases and their respective overlaps are shown, and the number of transcripts is indicated. The percentages of genes in each disease gene group that overlap with those of another disease are shown. Chi-square tests were used to compare transcript overlaps for each disease model with the two other diseases. *P*-values are shown for each comparison of overlap (between arrows); *p < 0.05; ***p < 0.0005; ****p<0.0001. **(B)** Heatmap of the 181 transcripts dysregulated in AD and EoE. **(C)** Heatmap of the 124 transcripts dysregulated in AA and EoE. **(D)** Heatmap of the 19 dysregulated probes common to the three diseases. NL, normal controls.

Comparing gene expression patterns in diseased tissues across the three diseases revealed the presence of a 5% overlap between AD and AA, a 10% overlap between AD and EoE, and a 7% overlap between EoE and AA (Figure 1A). The transcriptomic profile overlap between EoE and AD (181 transcripts or 10%) was significantly greater (*p* = 0.006) than that between EoE and AA (124 transcripts or 7%) (Figure 1B); these overlaps were based on presence or absence of dysregulation only, not a specific or common direction of the dysregulation. When genes dysregulated only in the same direction were evaluated, there was a significantly different overlap between EoE and AD (148 genes) than between EoE and AA (80 genes) (*p* < 0.0001) (Figure 1A). EoE and AD shared 148 genes (106 commonly upregulated + 42 commonly downregulated) of the initial 1,731 AD genes, whereas EoE and AA shared 80 genes (74 commonly upregulated + 6 commonly downregulated) of the initial 1,757 AA genes (Figures 1A–C). These results suggest that EoE and AD have a significantly greater gene expression overlap than EoE and AA.

### Common Inflammatory Genes Dysregulated in EoE, AD, and AA

Out of the common transcripts compared, 19 probe sets corresponding to 18 transcripts (1% of the total probes tested) were shared among all three diseases (Figure 1D). Ten of these genes were consistently upregulated in the three diseases, including histamine receptor H1 (*HRH1*), suppressor of cytokine signaling 3 (*SOCS3*), cathepsin C (*CTSC*), and plasminogen activator, urokinase receptor (*PLAUR*). However, the directions of the gene dysregulation were not similar in all the diseases. First, all 18 transcripts levels were upregulated in AA. Second, two genes were downregulated only in EoE (*UPP1* and *AKR1B10*) or only in AD (*CLDN1* and *SUSD2*) while being upregulated in the other two diseases. Finally, four genes were downregulated in both EoE and AD but not AA: filaggrin (*FLG)*, sciellin (*SCEL*), growth arrest-specific seven (*GAS7*), and diacylglycerol O-acyltransferase two (*DGAT2*) (Figure 1D). These results suggest that EoE, AD, and AA only share a small portion of their detectable transcriptomic signatures.

### EoE, AD, and AA Share a Similar Inflammatory Milieu but Differ in Epithelial Response

Ontology analysis revealed that the commonly upregulated genes were mainly involved in immune system processes in AD vs. EoE and AA vs. EoE, suggesting that the inflammatory component in the diseases is conserved and mostly upregulated across all three diseases (Table 1). Ontology analysis also highlighted that the genes involved in epidermal development were preferentially reduced in both EoE and AD (but this did not hold significance when corrected), whereas the genes involved in keratinocyte differentiation were upregulated in AA and downregulated in EoE, consistent with recent findings (51). Taken together, these results suggest that EoE shares common inflammatory components with both AD and AA but that the epithelial cell response may be divergent in the three diseases on the basis of the discordant direction of regulation of keratinocyte differentiation genes in EoE and AA and the absence of strong, significant enrichment between EoE and AD.

**Table 1:** Top shared biological processes involved.

**Term**	**Count**	**%**	***P*-value**	**Benjamini**
**106 concordant AD up EoE up**				
GO:0006508~proteolysis	18	18.56	***8.37E-05***	***5.90E-02***
GO:0002376~immune system process	17	17.53	***1.55E-04***	***3.10E-03***
GO:0022402~cell cycle process	11	11.34	***1.45E-03***	4.11E-01
GO:0007049~cell cycle	13	13.40	***1.54E-03***	3.11E-01
GO:0043632~modification-dependent macromolecule catabolic process	11	11.34	***1.64E-03***	2.57E-01
GO:0006955~immune response	12	12.37	***1.93E-03***	2.08E-01
**74 concordant AA up EoE up**				
GO:0002376~immune system process	16	25.81	***4.27E-06***	***8.11E-05***
GO:0006952~defense response	12	19.35	***2.52E-05***	***1.78E-02***
GO:0006968~cellular defense response	5	8.06	***1.00E-04***	***3.51E-02***
GO:0050896~response to stimulus	26	41.94	***7.23E-04***	***6.85E-03***
GO:0009611~response to wounding	9	14.52	***1.12E-03***	2.34E-01
GO:0006955~immune response	10	16.13	***1.50E-03***	2.35E-01
GO:0040011~locomotion	7	11.29	***6.55E-03***	***4.08E-02***
GO:0006954~inflammatory response	6	9.68	***9.38E-03***	7.40E-01
**42 concordant AD down EoE down**				
GO:0008544~epidermis development	4	11.43	***5.11E-03***	8.12E-01
GO:0007398~ectoderm development	4	11.43	***6.35E-03***	6.46E-01
GO:0007010~cytoskeleton organization	5	14.29	***9.06E-03***	6.28E-01
GO:0051704~multi-organism process	5	14.29	***3.50E-02***	4.73E-01
GO:0065003~macromolecular complex assembly	5	14.29	***3.66E-02***	7.81E-01
GO:0043933~macromolecular complex subunit organization	5	14.29	***4.49E-02***	8.10E-01
**37 discordant AA up EoE down**				
GO:0030216~keratinocyte differentiation	4	11.76	***2.36E-04***	6.69E-02
GO:0009913~epidermal cell differentiation	4	11.76	***3.06E-04***	***4.38E-02***
GO:0007398~ectoderm development	5	14.71	***4.51E-04***	***4.31E-02***
GO:0030855~epithelial cell differentiation	4	11.76	***1.99E-03***	1.35E-01
GO:0008544~epidermis development	4	11.76	***4.57E-03***	2.35E-01
GO:0006952~defense response	6	17.65	***4.76E-03***	2.08E-01
GO:0060429~epithelium development	4	11.76	***8.17E-03***	2.91E-01
**26 discordant AD down EoE up**				
GO:0016338~calcium-independent cell-cell adhesion	2	10.53	***2.41E-02***	9.99E-01
GO:0016337~cell-cell adhesion	3	15.79	***3.66E-02***	9.94E-01
GO:0009967~positive regulation of signal transduction	3	15.79	***4.13E-02***	9.80E-01

*Only biological processes with a significant p-value are shown. Significant enrichment is shown in bold italics*.

### IL-13–Stimulated Epithelial Cell Transcriptome Is Strongly Represented in Human EoE Compared to AD or AA

**Figure 2:**
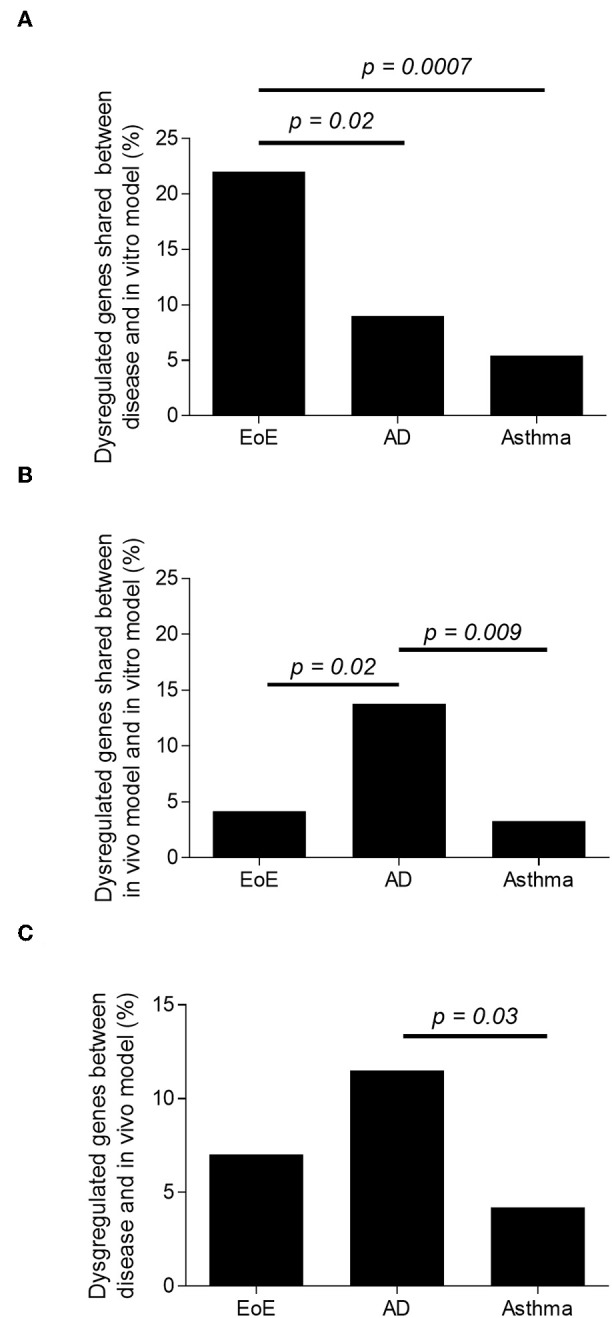
Representation of IL-13–induced, epithelial cell-derived genes in the human disease and in the *in vivo* models. Percentage of dysregulated transcripts in EoE, AD, and AA human diseases. **(A)** Percentage of dysregulated transcripts shared between EoE, AD, and AA human disease and IL-13–treated human primary esophageal, skin, and nasal epithelial cells *in vitro*, respectively. **(B)** Percentage of dysregulated transcripts shared between EoE, AD and AA mouse model and IL-13–treated human primary esophageal, skin, and nasal epithelial cells *in vitro*, respectively. **(C)** Percentage of dysregulated transcripts shared between EoE, AD, and AA human disease and EoE, AD, and AA mouse model, respectively.

In order to understand the global and specific involvement of IL-13 and epithelial cell response in the human EoE, AD, and AA, the three Th2 disease transcriptomes were compared separately with the transcriptomes of IL-13–stimulated primary epithelial cells isolated from the representative disease-affected tissues (esophagus, skin, or airway, respectively). The overlaps between the transcriptomes of the IL-13–stimulated epithelial cells and the respective diseases were 22% [as previously described (32)], 9%, and 5% for EoE, AD, and AA, respectively (Figure 2A and Supplementary Data File 1). The results demonstrated a significantly greater involvement of the IL-13–stimulated epithelial cell transcriptome in EoE than in AA (*p* = 0.0007) or AD (*p* = 0.02) transcriptomes. These results suggest that IL-13–dysregulated epithelial cell genes are strongly represented in EoE disease.

### IL-13–Stimulated Epithelial Cells Are Strongly Represented in the Murine AD Model

In order to understand the global and specific involvement of IL-13 in the murine EoE, AD, and AA transcriptomes, the three Th2 *in vivo* model transcriptomes were compared separately to the respective, IL-13–stimulated primary epithelial cells. We identified 4, 14, and 3% overlaps between the transcriptomes of the murine models of EoE, AD, and AA and the transcriptomes of their respective *in vitro* models (Figure 2B). When looking at the number of dysregulated transcripts overlapping, there was a significantly greater overlap of IL-13–stimulated epithelial response genes in the AD *in vivo* model than in the EoE (*p* = 0.023) or the AA (*p* = 0.009) *in vivo* models. These results do not take into consideration the directions of the dysregulations. For example, filaggrin was upregulated in the *in vivo* AD model transcriptome and downregulated in the *in vitro* AD transcriptome.

### The AD Murine Model Is Dominated by an IL-13–Induced Epithelial Cell Transcriptome and Is Representative of the Human Disease

**Figure 3:**
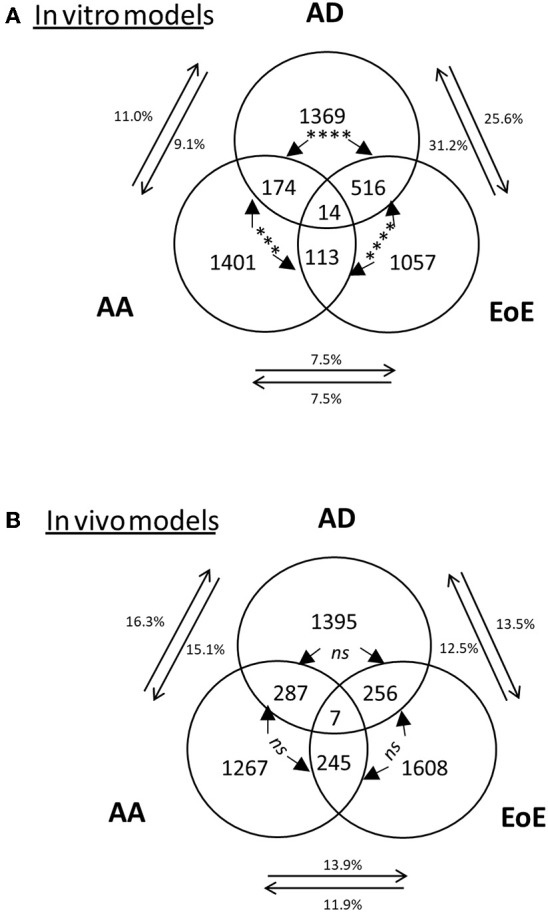
Representation of the dysregulated genes and overlaps among *in vitro* and *in vivo* models. Venn diagram of dysregulated transcripts in **(A)** *in vitro* models and **(B)** mouse models. The 3 amounts of dysregulated genes in EoE, AD, and AA in **(A)** *in vitro* models or **(B)** *in vivo* models and their respective overlaps are shown, with the number of transcripts indicated. The percentages of genes in each disease gene group that overlap with those of another disease are shown. Chi-square tests were used to compare transcript overlaps for each disease model with the two other disease models. *P*-values are shown for each comparison of overlap (between arrows); ***p < 0.0005;****p < 0.0001 “*ns”* is not significant.

The comparison of the *in vivo* models to their respective human diseases revealed 7, 12, and 4% overlap between the transcriptomes of the human disease and mouse model of EoE [as previously described (42)], AD, and AA, respectively (Figure 2C and the top gene list in Supplementary Table 1). Of note, the transcriptomes from several other murine models of AA and EoE were compared to the human diseases (data not shown). When the *in vitro* models were compared, a significantly increased (*p* < 0.0001) number of dysregulated genes overlapped between AD and EoE than between AA and EoE. A total of 26% of the transcriptome of the IL-13–stimulated primary skin epithelial cells overlapped with that of the IL-13–stimulated primary esophageal epithelial cells (Figure 3A). As a comparison, only 7.5% of the transcriptome of the IL-13–stimulated primary esophageal epithelial cells overlapped with that of the IL-13–stimulated primary airway epithelial cells (Figure 3A). The three *in vivo* models shared approximately 250 dysregulated genes (range 245–287, difference insignificant) when compared by pairs but shared only seven dysregulated genes when compared collectively (Figure 3B). Taken together, these data highlight the overlap between the skin and esophageal epithelial cell response to IL-13 and suggest that the AD model transcriptome is characterized by a large IL-13 epithelial cell-derived transcriptome.

## Discussion

This study demonstrates that gene dysregulation is more similar, in the amount of genes affected and in common direction, between EoE and AD than EoE and AA. The molecular overlaps, as measured by common gene expression profiles, among EoE, AD, and AA using multiple cellular sources and model systems are also reported. The transcriptome of IL-13–stimulated epithelial cells overlapped more with that of human EoE disease than human AD or AA. This study also identified that among the models tested, the mouse model of AD had greater transcriptomic overlap with its human disease counterpart than did mouse models of EoE or AA. These findings describe the first direct comparison of three Th2 diseases, identifying comprehensive sets of genes involved in multiple biological systems and providing insights into shared etiologic factors (and possible common therapeutic strategies) in allergic diseases.

A previous study using microarray analysis showed that IL-13, and more specifically IL-13–stimulated epithelial cell genes, are highly involved in the EoE disease transcriptome (32). In the study herein, the transcriptomic overlap for IL-13–induced epithelial cells with EoE (22%) was significantly different than the one observed with AD (9%) and AA (5%). Though these data reinforce the IL-13–induced epithelial cell transcript signature component in EoE, the relatively lesser importance of it in AD and AA is noteworthy. Within the greater perspective, the *in vitro* models utilize only a single stimulus (IL-13) and cell type (primary epithelial cells) and thus lack the biological complexity of whole tissue biopsies or brushes. Thus, the common inflammatory components resulting from the complex Th2 inflammation, infiltrating immune cells, and tissue remodeling are not represented in these analyses. A complex *in silico* reconstitution of the diseases with several *in vitro* models such as epithelial cell stimulated with other cytokines or other types of primary cells stimulation such as the recently described esophageal organoids (52) would allow deeper insights into the gene interactions among the different cell types and pro-inflammatory stimuli involved in these diseases. Eighteen transcripts were found dysregulated in the three diseases. Some of the genes, such as *HRH1, MMP19, SOC3, CISH*, and *FLG*, are known to be involved in inflammation and/or Th2 diseases. Interestingly, *PLAUR—*plasminogen activator, urokinase receptor—has been recently described to be involved in regulating a protease/anti-protease upstream pathway in EoE (53). Other genes, such as *SUSD2* and *DGAT2*, are not well-studied in Th2 inflammation, and their exact roles in EoE, AD, and AA disease pathogeneses and pathways have yet to be determined.

An interesting but conflicting finding comes from the comparison of the *in vivo* models with the IL-13–stimulated primary cells. Our results suggest a significantly higher involvement of IL-13 in the AD model than in the EoE or AA murine models. Other EoE and AA animal models were analyzed in parallel, including IL-13–induced AA and *Aspergillus fumigatus*–induced EoE (data not shown), and only the most relevant models are shown. In these CC10-rtta / Tet-on–IL-13 mice, the EoE disease results from the induction of IL-13. This led to our assumption that more IL-13–induced genes should have been found in this IL-13 driven EoE model than in the allergen-induced AD model (42). Additionally, other EoE and AA *in vivo* models have been characterized as IL-13–driven models. For example, intranasal injection of IL-13 or its overexpression in the lungs is sufficient to induce both allergic airway inflammation and esophageal eosinophilia (42, 54). The AD model involves repeated epicutaneous sensitization with an *Aspergillus fumigatus* extract, which induces a robust systemic Th2 response. However, the occurrence of the AD-like symptoms has been shown to be independent of IL-13/IL-4 and STAT6 (9, 10, 30), suggesting that IL-13 is involved in the molecular signature, rather than the skin symptom presence, of the *in vivo* AD model. These findings may reflect both the high involvement of IL-13 in the AD model molecular pathogenesis and the convergence of the human and mouse skin keratinocytes. Indeed, this high transcriptomic overlap was not conserved between the human AD disease and the *in vitro* model, suggesting that other mechanisms independent of IL-13 and keratinocytes have an important contribution in the AD disease transcriptome.

Transcriptomic studies in tissues are highly dependent on the type of studied tissue examined. For example, sampling lung tissue of patients with and without asthma is difficult. The tissues used to represent airway inflammation in this study were nasal epithelial cells re-stimulated with IL-13 compared to nasal brushes of patients with asthma. This approach is a non-invasive way to assess the molecular phenomenon involved in human asthma. Additionally, the relevance of nasal epithelial cell a surrogate for bronchial epithelial cell have recently been demonstrated in asthma with IL-13 stimulation by Roberts et al. (55). Murine lung tissue was used for the *in vivo* airway inflammation and transcriptomic analysis. Cross-species and tissue-to-cell transcriptomic analyses may identify fewer overlapping expression profiles than what would be expected with similar comparisons using human tissue and human *ex vivo* cells. Indeed, when an IL-13–stimulated, primary lung epithelial cell transcriptome was also analyzed (instead of nasal epithelial cells as presented in the analysis herein), the transcriptomic overlaps were similar (data not shown). Finally, the number of genes detected is limited by the technic used. Transcriptomic was performed using Affymetrix microarrays, RNA sequencing would have allowed detection of more genes with low levels of detection.

Comparative analyses of the transcriptomes from patient-derived samples from all three diseases revealed a stronger overlap between EoE and AD than between EoE and AA. The convergence of the skin and esophageal tissue toward squamous, stratified epithelia can explain most of this discrepancy because EoE shared inflammatory genes with both AD and AA but had concordance with AD and discordance with AA in regard to epithelial/epidermal or keratinization genes (e.g., claudin 1, filaggrin, small proline-rich proteins). The similarity of the cellular infiltrates may explain, at least in part, the strong overlap observed with inflammatory genes. Of the overlap in gene expression, 5–10% was observed when comparing the different diseased human tissues. Indeed, in other studies in which the same tissue types were compared between different diseases, such as Crohn's disease and ulcerative colitis (56) or psoriasis and AD (57), the percentage overlap approached 24%, supporting the importance of the nature of the tissue in the transcriptomic overlaps. In this study, in addition to the different tissue comparisons, the number of genes identified for the analysis was also smaller because of more stringent gene selection criteria, which may contribute to the reduced percentage of overlapping transcripts between models. Our goal was not to find candidate genes but rather to have a global overview of the common molecular signatures among the three diseases. In addition, the technical limitations of microarray analyses may skew results and interpretation. For example, interleukins are typically undetected in microarrays yet are differentially expressed when quantitative PCR is performed (58, 59). Thus, cytokines suspected to be dysregulated in the three diseases (e.g., IL-4, IL-13, and TSLP), were not detected and therefore not shown as conserved in the *in vivo* models and the diseases. Our study is thus limited to comparing expression changes in highly expressed (detectable) and/or highly dysregulated genes and detected with the same probe. Indeed, we took a novel approach examining nine different biological systems (three diseases, three *in vivo* models, and three *in vitro* models) via deep transcriptomics. In addition to identifying common genes and their degree of dysregulation across the nine systems, this study also highlights the relevance of the *in vitro* and *in vivo* models to their respective human diseases.

In conclusion, we have described the transcriptomic similarities and differences of three Th2 diseases across a variety of model systems. Our results substantiate the central role of common epithelial responses in these Th2-associated diseases (51) and draw increased attention to the relative sharing of disease mechanisms between AD and EoE. We identified the conserved genes in the three diseases and provided a comprehensive set of genes specific to one tissue, model, or species. This global analysis of diseases and comparison to simplistic *in vitro* models could be extended to other cytokine cell stimulations and/or hematopoietic cell transcriptomes in efforts to model disease transcriptional changes *in silico* and to uncover key pathways for targeted therapy.

## Data Availability Statement

The datasets generated for this study can be found in the published literature (32) or obtained from public databases (www.ncbi.nlm.nih.gov/pubmed) GEO repository [GSE32924 (47) and GSE19187].

## Ethics Statement

Ethical review and approval was not required for the study on human participants in accordance with the local legislation and institutional requirements. Written informed consent for participation was not required for this study in accordance with the national legislation and the institutional requirements. The animal study was reviewed and approved by SCAV—Affaires vétérinaires Ch.Des Boveresses 155, 1066 Epalinges.

## Author Contributions

CB conceived the research hypothesis. CB, SH, and RD-L performed the experiments. FF, SH, RD-L, CB, and FR analyzed the data. FR and JS verified the analytical methods. MR and GK lead the clinical work. RD-L, CB, JS, GK, and MR draft the manuscript. All authors reviewed the results and contributed to the final manuscript.

### Conflict of Interest

The authors declare that the study received funding from Société des Produits Nestlé SA. The employees of the funders were involved in the design, the data analysis, the drafting of the manuscript and the decision to publish the work.

## Abbreviations

AA: Allergic airways
AD: atopic dermatitis
*CTSC*: cathepsin C
CLDN1: claudin 1
EoE: eosinophilic esophagitis
FDR: false discovery rate
*FLG*: filaggrin
*HRH1*: histamine receptor H1
*PLAUR*, plasminogen activator: urokinase receptor
*SOCS3*: suppressor of cytokine signaling 3
TEWL: transepidermal water loss.
