# Supplementary figures and images for: Transcriptomic Analysis Links Eosinophilic Esophagitis and Atopic Dermatitis
Source: Front Pediatr. 2019 Nov 20;7:467. doi: 10.3389/fped.2019.00467 (PMC6879454; doi:10.3389/fped.2019.00467)

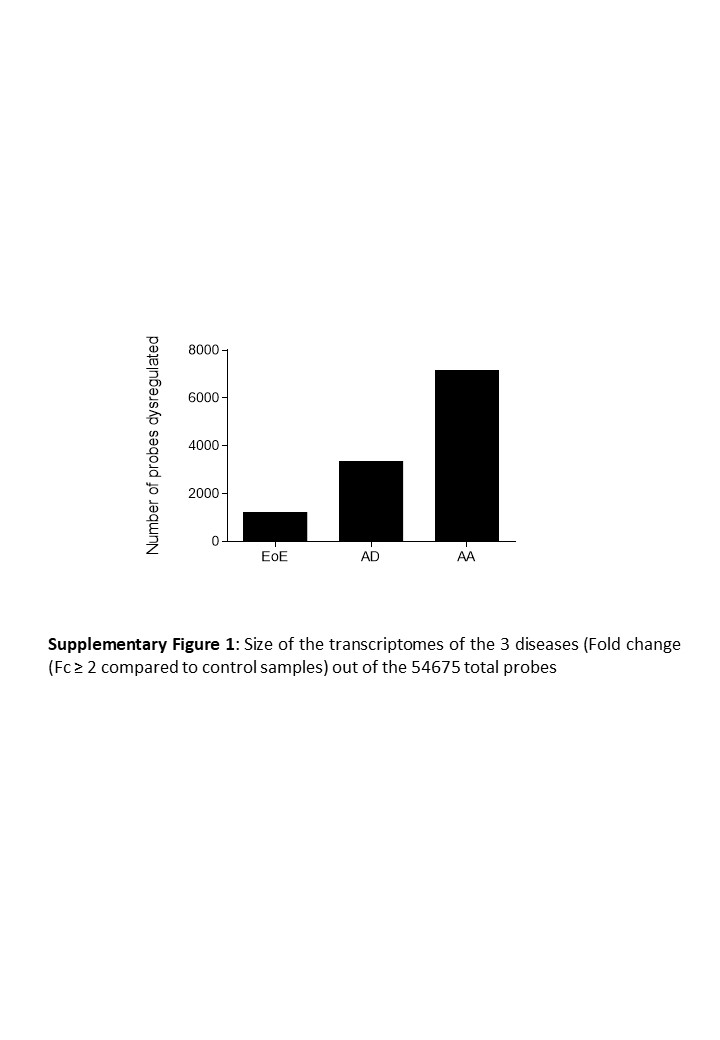

Supplement: Supplementary file 2 [file Image_1.JPEG]
